# Vitamin D Intake and Serum 25-Hydroxyvitamin D Levels in Korean Adults: Analysis of the 2009 Korea National Health and Nutrition Examination Survey (KNHANES IV-3) Using a Newly Established Vitamin D Database

**DOI:** 10.3390/nu8100610

**Published:** 2016-09-29

**Authors:** Kyoungok Yoo, Jinah Cho, Sunyung Ly

**Affiliations:** Department of Food and Nutrition, Chungnam National University, Daejeon 305-764, Korea; 68ykok@daum.net (K.Y.); jacho@cnu.ac.kr (J.C.)

**Keywords:** vitamin D intake, serum 25-hydroxyvitamin D, food sources, Korean adults

## Abstract

Vitamin D is important for maintaining bone health and may prevent various diseases (i.e., cardiovascular disease and cancer). The aim of this study was to estimate vitamin D intakes of Korean adults using the Korea National Health and Nutrition Examination Survey (KNHANES, 2009) data and a newly established vitamin D database. KNHANES (2009) participants (*n* = 4541; 2021 men; 2520 women) aged ≥20 years were included. Dietary vitamin D intake, serum 25-hydroxyvitamin D (25(OH)D), and the relationship between vitamin D intake and serum 25(OH)D were evaluated. In men and women, vitamin D intakes were 4.00 ± 0.17 µg/day and 2.6 ± 0.1 µg/day respectively, and serum 25(OH)D concentrations were 19.78 ± 0.33 ng/mL and 17.10 ± 0.26 ng/mL respectively. Serum 25(OH)D concentrations of men aged <50 years and women aged >20 years were under 20 ng/mL. After adjusting for confounding factors, the positive relationship between vitamin D intake and serum 25(OH)D was observed in total subjects (*p* < 0.05), excluding participants ≥50 years old. The main food sources for vitamin D among Korean adults were fish/shellfish (71.34%) and egg (14.89%). Korean adults should increase their serum 25(OH)D concentrations by increasing vitamin D intake.

## 1. Introduction

Vitamin D is widely known to be involved in the homeostasis of calcium and phosphate and the maintenance of skeletal health. In recent studies, vitamin D has been inversely associated with the risk of various diseases, such as obesity and hypertension, various cancers, cardiovascular disease, type 2 diabetes, viral respiratory infections, tuberculosis, meningitis, Alzheimer’s disease, and Parkinson’s disease [1,2,3,4]. Furthermore, increased serum vitamin D levels are associated with a significant reduction in morbidity and mortality [5].

Although serum 25-hydroxyvitamin D (25(OH)D) concentrations are an indicator of adequate vitamin D levels in the body [6], a worldwide standard serum 25(OH)D level indicative of vitamin D deficiency has not been determined. According to the Institute of Medicine [7] and a majority of related publications, a serum 25(OH)D concentration of 20 ng/mL is considered deficient because even a serum 25(OH)D concentration of 50 nmol/L (20 ng/mL) was found to have no positive effects on calcium absorption and bone density [8,9]. When vitamin D deficiency was defined by a serum 25(OH)D concentration of <20 ng/mL, the global vitamin D deficiency rates were found to be >40% among the elderly and women in the United States (US) and Europe and >50% among children and adolescents with insufficient sun exposure in Saudi Arabia, Turkey, and Lebanon [10,11,12]. In South Korea, vitamin D deficiency was also found in 47.3% of males aged ≥10 years and 64.5% of females aged ≥10 years according to data from the Korea National Health and Nutrition Examination Survey (KNHANES) in 2008 [13].

Food sources of vitamin D are limited (i.e., seafood, eggs, milk and dairy products, meats, and mushrooms), and thus, vitamin D is synthesized in the body primarily through ultraviolet exposure. Therefore, the importance of food sources of vitamin D has not been highly emphasized. However, in modern society, the amount of time spent outdoors has gradually decreased; moreover, the American Cancer Society recommends the use of sunblock for skin care (i.e., anti-aging) and for preventing skin cancer [14]. As a result, modern serum 25(OH)D levels have been reported to be poor [15]. Higher intakes of fatty fish and cod liver oil were found to be responsible for the higher serum vitamin D concentrations in people from Norway and Sweden (countries with insufficient sunlight exposure) compared to people from Spain and Italy [11]. In addition, a higher intake of vitamin D-rich food (i.e., fish/shellfish and eggs) among Japanese people was associated with higher serum vitamin D concentrations during November (a time of year with less sun exposure) [16]. Therefore, it is necessary to increase the intake of vitamin D-rich foods in order to maintain optimal serum 25(OH)D concentrations.

In South Korea, vitamin D was not included in the food composition databases used for the KNHANES, and therefore, vitamin D intakes among Korean people have not yet been assessed. In this study, we intended to supplement the vitamin D food composition table of the largest vitamin D database in Korea published by the Korean Nutrition Society by adding data regarding vitamin D-containing foods, which are available in domestic and international institutions, including Japan and the US. The established data were used in the analysis of KNHANES (2009) to assess the vitamin D intakes of Korean adults aged ≥20 years. Furthermore, we aimed to identify food sources that contribute to high dietary vitamin D levels in Korean people and to evaluate the vitamin D levels required to maintain adequate serum 25(OH)D concentrations.

## 2. Results

### 2.1. General Characteristics

Approximately 28.1% of the 4541 participants (2021 men; 2520 women) were smokers. Compared to women, a higher percentage of men (47.0%) were smokers (5.9%; *p* < 0.001). Likewise, more men (75.9%) than women consumed alcohol (41.0%; *p* < 0.001). The percentage of participants with high physical activity was significantly higher in men (22.1%) than in women (15.2%; *p* < 0.001). A higher percentage of men (34.0%) had >5 h of sun exposure compared to women (16.1%; *p* < 0.001). However, the percentage of participants who used vitamin/mineral supplements was lower in men (17.1%) than in women (24.3%; *p* < 0.001; Table 1).

### 2.2. Energy and Vitamin D Intake and Serum 25(OH)D Concentrations

The highest energy intake was observed in Korean men aged 30–49 years and women aged 20–29 years, and the lowest energy intake was observed in men and women aged ≥75 years (*p* < 0.001). The mean daily vitamin D intake before adjusting for energy intake was 3.76 ± 0.19 µg/day in men and 2.56 ± 0.10 µg/day in women. After adjusting for energy intake, the mean daily vitamin D intake increased to 4.00 ± 0.17 µg/day in men and 2.64 ± 0.10 µg/day in women. The mean daily vitamin D intake before adjusting for energy intake was found to be the highest in men and women aged 30–49 years and lowest in those aged ≥75 years (men, *p* < 0.001; women, *p* < 0.01); there was no significant difference in the mean daily vitamin D intake after adjusting for energy intake between age groups. The percentage of men and women with less than the adequate intake (AI) of vitamin D was 74.6%–95.9% and 84.5%–96.3%, respectively. Therefore, a considerable number of participants of both genders did not have AIs of vitamin D. The mean serum 25(OH)D concentration was 20 ng/mL (the cut-off point for deficient serum 25(OH)D concentration in this study) in men aged <50 years and >20 ng/mL in men aged ≥50 years. In particular, the serum 25(OH)D concentration in men was the highest for those aged ≥75 years (21.65 ± 0.80 ng/mL) and lowest for those aged 20–29 years (16.43 ± 0.50 ng/mL) (*p* < 0.001). Mean serum vitamin D concentrations of women in Korea were <20 ng/mL, and the serum vitamin D concentrations in women were significantly lower than those in men (*p* < 0.001). The percentages of men and women with deficient serum 25(OH)D concentrations were 40.9%–76.8% and 62.7%–86.1%, respectively (Table 2).

After adjusting for confounding factors (age, drinking status, smoking status, physical activity status-high, sun exposure time and use of vitamin/mineral supplements), there was a positive relationship between vitamin D intakes and serum 25(OH)D concentrations in total subjects and all participants <50 years old (*p* < 0.05). However, the influence of these was very low with *R*^2^-value of 0.089 and 0.072, respectively. There were no significant relationships between vitamin D intakes and serum 25(OH)D concentrations in participants aged ≥50 years (Table 3).

### 2.3. Food Sources of Vitamin D

The fish and shellfish food group was the highest contributor to vitamin D intake in both men and women, followed by eggs, milk and dairy products, meat and meat products, mushrooms, and grain and grain products. The vitamin D intake from fish and shellfish was significantly higher in men (2.81 ± 0.17 µg/day; 71.69%) than in women (2.02 ± 0.10 µg/day; 62.35%; *p* < 0.001). Furthermore, vitamin D intake from milk and dairy products was lower in men (0.16 ± 0.02 µg/day; 4.12%) than in women (0.24 ± 0.02 µg/day; 7.41%; *p* < 0.05). Compared to men, women were observed to have higher vitamin D intake from mushrooms, but this finding was not significant (Table 4).

Anchovies were found to have the highest contribution towards the vitamin D intake in Korean adults (23.58% in men; 23.91% in women). The total contribution of anchovy, mackerel, and egg towards the vitamin D intake in both men and women was >50%. In addition to fish and shellfish, the main food sources of vitamin D were milk (3.07%), pork (1.79%), and mushroom (2.46%) in men, and milk (5.59%), mushroom (3.95%), and pork (1.72%) in women (Table 5).

## 3. Discussion

In the present study, the dietary vitamin D database established by the Korean Nutrition Society was used to supplement the vitamin D intake assessment in Korean adults. In the newly established vitamin D database, coverage for the number of related foods increased from 34.1% to 45.4%. However, the overall coverage was not high because vitamin D is only present in a limited number of foods, and the coverage also contains vegetables without any vitamin D. However, when the vitamin D intakes were assessed in the present study, the coverage for vitamin D-containing foods in the food database for Koreans was found to be >95%, and therefore, the newly established database was considered feasible for effectively calculating vitamin D intakes.

The mean daily vitamin D intake after adjusting for energy intake was 1.36 ± 0.07 µg/day higher in men (4.00 ± 0.17 µg/day) than in women (2.64 ± 0.10 µg/day). The mean daily vitamin D intake was the highest in those aged 30–49 years for both genders and was the lowest in those aged ≥75 years. Furthermore, vitamin D intake after adjusting for energy intake was slightly decreased in participants aged <50 years, but increased in those aged ≥50 years. Based on these results, those aged ≥50 years should consume a more vitamin D-dense diet. However, the mean intake in those aged <50 years was below the AI, suggesting that vitamin D should also be increased in the diet of this group.

The mean vitamin D intakes in American men and women aged 25–74 years in 2007–2009 were 6.15 ± 0.27 µg/day and 4.53 ± 0.16 µg/day, respectively; these intake values are higher than those observed in Koreans. However, when assessed by age groups, the lowest vitamin D intake in America was observed in both men and women aged 25–39 years, and the highest vitamin D intake was observed in women aged 40–54 years and men aged 55–74 years [17]. The mean vitamin D intakes in Finnish men and women aged 25–65 years were 7.1 µg/day and 5.2 µg/day, respectively, while the mean vitamin D intakes in men and women aged 65–74 years were 9.0 µg/day and 6.5 µg/day, respectively [18]. According to the results of the National Diet and Nutrition Survey published in 2012 in Japan, the mean vitamin D intake in men and women aged ≥20 years was 8.3 ± 9.7 µg/day and 7.2 ± 8.2 µg/day, respectively [19], which was higher than intake in men and women in South Korea. However, according to the National Diet and Nutrition Survey in 2008–2012 in the United Kingdom (UK), the mean daily vitamin D intake in men and women aged 19–64 years was 3.0 ± 2.2 µg/day and 2.4 ± 1.8 µg/day respectively, which was lower than that of men and women in South Korea [20].

In accordance with the 2010 Dietary Reference Intakes for Koreans, the Korean Nutrition Society established the AI for vitamin D at 5 µg/day in both men and women aged 20–49 years and 10 µg/day in those aged ≥50 years. In this present study, the 30–49-year age group was observed to have the lowest percentage of participants who consumed less than the AI for vitamin D (men, 74.6%; women, 84.5%). Approximately 90.3%–95.9% of men and 93.8%–96.3% of women aged ≥50 years consumed less than the AI of vitamin D for Koreans; these percentages were higher compared to those of adults aged <50 years. This trend can be observed in other countries. The percentages of Brazilian men and women aged ≥65 years who consumed less than the AI (10 µg/day) for vitamin D were 98.8% and 99.0%, respectively, which were higher than the percentages of Korean men and women [21]. Additionally, 90.2% of German elders aged 66–96 years consumed less than the AI (10 µg/day) of vitamin D [22]. According to these findings, the elderly in South Korea and foreign countries do not meet the daily dietary reference vitamin D intakes. The energy requirement for elders is lower than that for younger adults, and therefore, dietary intake decreases; however, the AI of vitamin D is higher for the elderly in order to prevent diseases and fractures due to a decline in subcutaneous vitamin D synthesis capacity [23,24].

We found that dietary intake in the elderly was low, and it might be difficult to meet the currently set reference AI of vitamin D (10 µg/day) through food intake only. Therefore, there is an increased need for foods fortified with vitamin D. In Japan, the serum 25(OH)D concentration was found to increase from 23.1 ng/mL to 36.0 ng/mL in healthy female college students living in dormitories after the consumption of 180 mL of milk fortified with 2 µg of vitamin D for 8 weeks [25]. In the UK, the percentage of at-risk groups estimated to have vitamin D intakes below the UK Reference Nutrient Intakes was reduced from 93% to 50% after consuming a simulated fortification of 10 µg vitamin D/100 g wheat flour, and no participants exceeded the UK Tolerable Upper Intake Level; the 2.5th percentile of the population winter serum 25(OH)D concentration increased from 20 to 27 nmol/L after fortification [26]. The serum 25(OH)D concentrations in American participants aged 18–79 years who consumed orange juice fortified with 1000 IU of vitamin D_3_ and 1000 IU of vitamin D_2_ increased to 12.8 ± 10.1 ng/mL and 10.6 ± 7.2 ng/mL, respectively [27]. In Finland, vitamin D has been added to fluid milk and dairy products as well as margarine and butter since 2003. As a result, the serum 25(OH)D concentrations increased by 50%, and vitamin D insufficiency decreased by 50% (78% in January 2003 to 35% in January 2004) [28]. Therefore, there is a need for increased consumption of foods fortified with vitamin D in Korea. About 2–2.25 µg/180 mL of vitamin D is added to high-calcium milk products and 0.75–1.9 µg/190 mL of vitamin D_3_ is added to soy milk products in South Korea, and therefore, continued consumption of these fortified foods is expected to increase serum 25(OH)D concentrations.

Older adults may have decreased sun exposure, reduced dermal conversion of 7-dehydrocholesterol to vitamin D_3_, and secondary hyperparathyroidism, which contribute to poor serum 25(OH)D concentrations [29]. However, in the present study, the serum 25(OH)D concentration in Korean adults aged ≥50 years was found to be higher than that of adults aged <50 years. Similar findings were observed in the KNHANES (2008) [13]. In addition, vitamin D status was found to improve with aging in the Thailand National Health Examination Survey (2008–2009) [30]; this was explained by the fact that the elderly had more leisure and outdoor activity time. Therefore, after adjusting for sun exposure time, age, drinking status, smoking status, physical activity (high), and use of vitamin/mineral supplements regarded as a confounding factor for serum 25(OH)D concentrations, vitamin D intake in all participants and those aged <50 years was positively related to serum 25(OH)D concentration in this study. However, the influence of these was very low. This finding may differ by age group. It can be concluded that an increase in dietary vitamin D intake for young Korean adults with low serum 25(OH)D concentrations can contribute to an increase in serum 25(OH)D concentration.

Black et al. [31] described how an intake of 1 µg/day of vitamin D-fortified foods could increase serum 25(OH)D concentrations by 1.2 nmol/L. Cranney et al. [32], found that every 2.5 µg of vitamin D_3_ increased serum 25(OH)D concentration by 1–2 nmol/L; the increase in serum 25(OH)D concentration could be greater when the initial serum 25(OH)D concentration is low and the study period is longer. These findings are consistent with the results of the present study. If the findings of the study by Cranney et al. [32] are applied to Koreans, a 5 µg/day increase in vitamin D intake among Korean adults aged <50 years can be expected to additionally increase serum 25(OH)D concentration by 1.6–2.4 ng/mL, which can help prevent vitamin D deficiency (<20 ng/mL or <50 nmol/L). In a study by Cashman et al. [33], a vitamin D intake of 9 µg/day on average is needed to achieve a serum 25(OH)D concentration of 50 nmol/L. Considering the dietary reference nutrient intakes for Koreans, the AI for vitamin D in young Korean adults should be >10 µg, which may be suitable in order to augment serum 25(OH)D concentration to >20 ng/mL.

In order to increase vitamin D intakes among Koreans, it was necessary to evaluate the propensity for consumption of food sources of vitamin D. The main food sources of vitamin D included fish, eggs, meats, milk, mushrooms, and grains. Among these food sources, fish and shellfish had the highest contribution towards the total vitamin D intake (>70% in both men and women). The main food sources of vitamin D in the world depend on the dietary habits of people and the nutrition policies in each country. The main sources of vitamin D for Americans and Canadians were milk, followed by meat and fish [34], and the contribution of vitamin D-fortified milk towards vitamin D intake was very high in American men (58%) and women (39%) [35]. Similar results were observed in Finland [36]. The contribution of low-fat milk and dairy products generally fortified with vitamin D was found to be high in Sweden [37]. However, the highest contributors of food sources for vitamin D intake in the UK were meat and meat products followed by fat spreads, cereals and cereal products, and fish and fish dishes [20]. In Germany, the main food sources of vitamin D were fish, fats and oils, milk, and breads [22], and in Japan, the main sources of vitamin D were reported to be fish, followed by eggs and mushrooms [38]. Fish and shellfish contribute to over 70% of vitamin D intake in Korea, while they contribute to about 38.5% in Germany [22], 15%–18% in the UK [39], 38% in France [40], 65% in Spain [41], and 12% in Ireland [42]. Japan was shown to be similar to Korea with respect to the contribution of fish and shellfish towards vitamin D intake (79%) [43]. In particular, the contribution of fish and shellfish towards vitamin D intake in the Japanese elderly has been reported to be very high (90.7%) [43], which was similar to Korean elders. In a Japanese study, the serum 25(OH)D concentration in elderly women who consumed fish ≥4 times/week was 10.1 nmol/L (4.04 ng/mL) higher than that of elderly women who consumed fish 1–3 times/week [38]. Increasing fish intake is considered to be the easiest way to increase dietary vitamin D intake. Anchovy, one of the main food sources of vitamin D for Korean people, is a good food source of vitamin D (4 µg/100 g), but has a high sodium content (240 mg/100 g). Therefore, caution is required with respect to anchovy consumption, including reducing the intake of water-soluble sodium in soup. Furthermore, an increased intake of excellent food sources of vitamin D, such as milk and eggs, is recommended. However, it may be difficult for the elderly to easily increase intake of such foods due to lactose intolerance [44] or cardiovascular disease. Therefore, consumption of soy milk fortified with vitamin D and vitamin D supplements can be considered an alternative solution. Furthermore, it is necessary to increase the intake of vitamin D among Korean people by introducing national food fortification policies in South Korea, which have already been implemented in the US and Finland. It is not easy to develop dietary reference intakes for vitamin D collectively because sunlight exposure differs depending on lifestyle and residential area.

The Ministry of Health, Labour and Welfare of Japan established the daily reference intake for vitamin D based on a sunlight exposure time needed to synthesize 5.5 µg of vitamin D [45]. According to that study, sunlight exposure for about 2 h has been shown to synthesize about 7.5 µg of vitamin D_3_ among those in the Sapporo, Tsukuba, and Okinawa regions. These findings seem to have been used in calculating the adequate dietary reference intakes of vitamin D for Korean people.

This study had a few limitations. Although the intakes of vitamin D supplements were assessed to determine the total daily intake of vitamin D, only the intake or non-intake of vitamin/mineral supplements was surveyed in the KNHANES (2009). Therefore, there are no available data regarding the intake amount and duration of single vitamin D supplements, and our evaluation of the vitamin D intakes in Korean people may thus be limited. In addition, the radioimmunoassay method used to assess serum 25(OH)D concentrations has been reported to underestimate serum 25(OH)D concentrations more than liquid chromatography-tandem mass spectrometry method [46]. Recently, liquid chromatography-tandem mass spectrometry method has become a gold-standard method for assessing serum 25(OH)D concentrations. In future studies, this method should be considered also. Nevertheless, the vitamin D intakes in the present study were calculated by using reliable data from Korean adults, which can assist in evaluating optimal vitamin D intake and developing dietary reference intake of vitamin D for Korean people.

## 4. Materials and Methods

### 4.1. Study Data and Design

**Figure 1:**
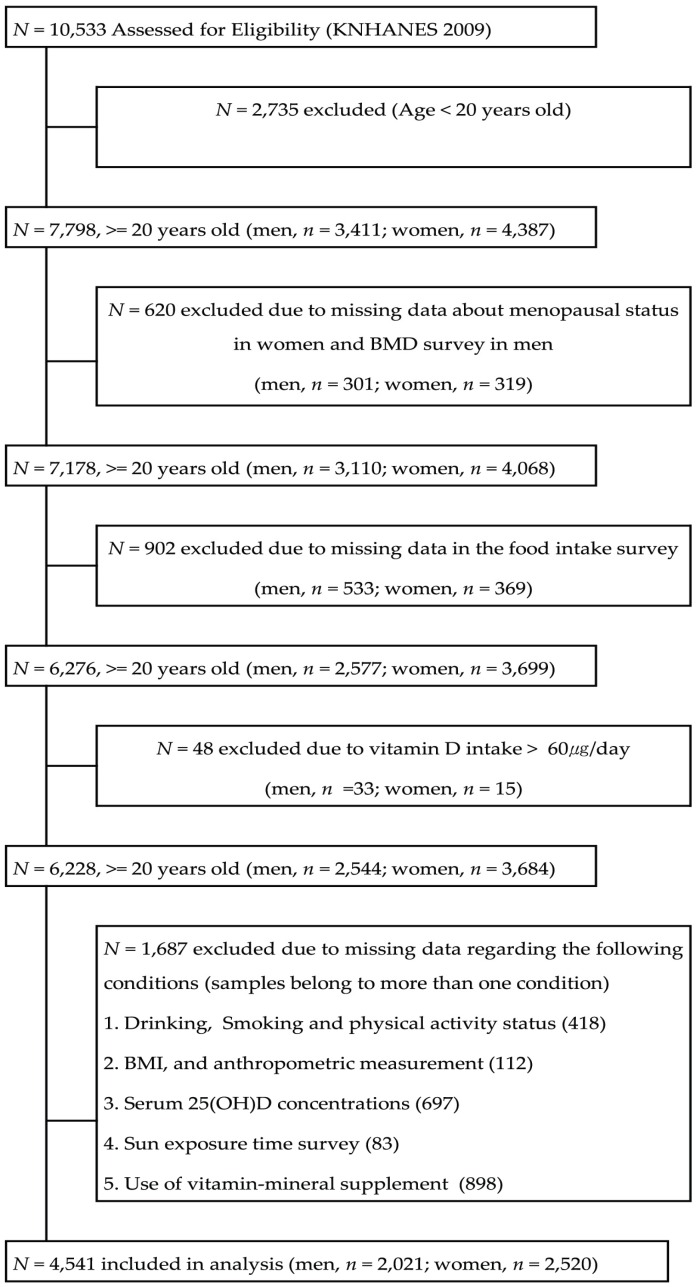
Flowchart of subject inclusion and exclusion in the Korea National Health and Nutrition Examination Surveys 2009 (BMD, bone mineral density; BMI, body mass index; 25(OH)D, 25-hydroxyvitamin D).

This study was based on cross-sectional data obtained from the KNHANES (IV-3) in 2009. The KNHANES (IV-3) contains a basic database with information from health interviews, biochemical tests, and a nutrition survey. The health interviews involved personal interviews and self-administered questionnaires. The biochemical tests were conducted by direct anthropometric measurements and blood analyses. The nutrition survey was conducted by trained dieticians in 3975 households from 200 Korean national districts. Of the 10,533 participants, 7798 participants (3411 men; 4387 women) aged ≥20 years were evaluated. Participants with unknown menopausal status (*n* = 620); missing data in the food intake survey (*n* = 902); vitamin D intake > 60 µg/day (*n* = 48); and missing data regarding their status of drinking, smoking, physical activity, body mass index and anthropometric measurements, serum 25(OH)D concentrations, sun exposure, and use of vitamin/mineral supplements (*n* = 1687) were excluded from this study. Finally, data from 4541 participants (2021 men; 2520 women) were used in this analysis (Figure 1). The participants were divided into the following 5 groups based on age (i.e., 20–29 years, 30–49 years, 50–64 years, 65–74 years, and >75 years) and 2 groups by gender (i.e., male and female). The study protocol was approved by the ethical committee of Chungnam National University, Korea (201406-SB-029-01).

### 4.2. Vitamin D Database

Based on the vitamin D database (1191 foods) in ‘Food Values 2009’ published by the Korean Nutrition Society [47], a vitamin D database for 397 foods was newly established using data from the Food Composition Table (7th revision, 2006; published by the National Rural Living Science Institute, Korea; 104 foods) [48], the Functional Food Composition Table (2009; published by the National Academy of Agricultural Science, Korea; 20 foods) [49], Standard Tables of Food Composition in Japan (5th revision; published by the Japan Association of Training College For Cooks; 131 foods) [50], and the vitamin D (D_2_ ± D_3_; mcg) content of selected foods based on the United States Department of Agriculture National Nutrient Database for Standard Reference (release 23; 142 foods) [51]. If the vitamin D content in the food was different depending on the data source, the final data were cited based on the following order: The Korean Nutrition Society, the National Rural Living Science Institute, the National Academy of Agricultural Science, Japanese data, and US data. However, if data regarding foods that were indicated as ‘trace’ in the Korean Nutrition Society data and other data sources were also available, we cited the latter. Our final vitamin D database consisted of 1588 foods. The coverage for the number of foods in the newly established vitamin D database was 45.4%. Contents of dietary supplements were not documented in the KNHANES (2009).

### 4.3. General Characteristics

Information regarding smoking status (yes/no), drinking status (yes/no), physical activity status (high: At least 20 min of vigorous intensity exercise 3 times a week, yes/no; mid: At least 20 min of moderate intensity exercise 5 times a week, yes/no; walking: At least 30 min of walking 5 times a week, yes/no), sun exposure time (<5 h or >5 h), and use of vitamin/mineral supplements (yes/no) were collected during the health interview.

### 4.4. Energy and Vitamin D Intake, Main Food Source of Vitamin D, and Serum 25(0H)D Concentrations

Daily intakes of energy and vitamin D in Korean adults were assessed using food intake data collected using a 24 h dietary recall questionnaire and the newly established vitamin D database. After adjusting for energy intake, we examined vitamin D intake and percentages of the participants who consumed less than the adequate intake (AI) of vitamin D. Serum 25(OH)D concentration was measured by radioimmunoassay (25-Hydroxyvitamin D 125I RIA kit; DiaSorin Inc., Stillwater, MN, USA) and a gamma-counter (1470 WIZARD; PerkinElmer, Turku, Finland). The prevalence, by gender and age, of a deficient serum 25(OH)D concentration (<20 ng/mL or <50 nmol/L) in participants was estimated. The cut-off point for a deficient serum 25(OH)D concentration in this study was 20 ng/mL (50 nmol/L), according to the study by Dawson-Hughes et al. [52]. After adjusting for confounding factors (age, drinking status, smoking status, physical activity status-high, sun exposure time and use of vitamin/mineral supplements), we evaluated the relationship between vitamin D intake and serum 25(OH)D concentrations. We calculated vitamin D intake and the contribution of main food groups by gender. Also, individual food sources and contribution of them were estimated by gender.

### 4.5. Statistical Analyses

Analysis of KNHANES data for weighting was performed by complex samples analysis according to the statistical guidance of the Korea Center for Disease Control and Prevention. The general characteristic of the participants, such as smoking, drinking, physical activity status (high, mid, walking), sun exposure time, and use of vitamin/mineral supplements were compared across the genders using the complex samples analysis. The differences in smoking, drinking, physical activity status (high, mid, walking), sun exposure time, and use of vitamin/mineral supplements by gender were analyzed using chi-square test in complex samples analysis. Means and standard errors of energy, vitamin D intakes by adjusted energy, and serum 25(OH)D concentrations were determined based on age and gender. Differences among groups were analyzed using descriptive statistics in complex samples general linear model (CSGLM). The relationship between vitamin D intakes and serum 25(OH)D concentrations in all participants and men and women aged ≥50 years and <50 years was analyzed using the simple linear regression analysis in complex samples analysis after adjusting for confounding factors (age, drinking status, smoking status, physical activity status-high, sun exposure time and use of vitamin/mineral supplements). Vitamin D intake by food groups was expressed as the mean and standard error after adjusting for energy intake using descriptive statistics in CSGLM. All statistical analyses were conducted using SPSS version 22.0 (SPSS Inc., Chicago, IL, USA). An alpha level of 0.05 was used to determine a significant *F*-value.

## 5. Conclusions

In South Korea, reliable and representative intakes of vitamin D in Korean people have not been investigated until quite recently. Therefore, we assessed the KNHANES (2009) results by supplementing the vitamin D database with Korean food composition data and determined the effects of vitamin D intake on serum 25(OH)D concentrations in Korean people. In the present study, serum 25(OH)D concentrations were deficient (<20 ng/mL) in adults aged <50 years, and the mean daily intake of vitamin D did not substantially meet the reference AI of vitamin D. However, it was found that an increase in vitamin D intake could increase serum 25(OH)D concentration because there was a positive relationship between vitamin D intake and serum 25(OH)D concentrations overall and in all participants aged <50 years. Therefore, the prevalence of vitamin D deficiency among Koreans can be potentially reduced by increasing fish and shellfish intake, consumption of fortified foods, and time spent outdoors.

## Figures and Tables

**Table 1:** Characteristics of participants.

Variables	Total	Men	Women	X^2^-Value
	(*n* = 4541)	(*n* = 2021)	(*n* = 2520)	
Smoking: Yes	28.1 (995)	47.0 (864)	5.9 (131)	943.75 ***
Drinking: Yes	59.8 (2434)	75.9 (1467)	41.0 (967)	569.64 ***
Physical activity (high) *:, Yes	18.9 (773)	22.1 (412)	15.2 (361)	34.36 ***
Physical activity (mod.) *: Yes	14.4 (678)	15.3 (314)	13.3 (364)	3.41
Physical activity (walking) *: Yes	45.6 (2062)	46.8 (972)	44.2 (1090)	3.13
Sun exposure time, >5 h	25.7 (1320)	34.0 (796)	16.1 (524)	188.32 ***
Vitamin/mineral supplements: Yes	20.4 (923)	17.1 (335)	24.3 (588)	35.476 ***

All variables are expressed as percentages (numbers). * Physical activity (high), vigorous intensity physical exercise for at least 20 min 3 times a week; Physical activity (mod.), moderate intensity physical exercise for at least 20 min 5 times a week; Physical activity (walking), walking for at least 30 min 5 times a week. *** Chi-square test *p*-value < 0.001 for differences between men and women.

**Table 2:** Mean energy and vitamin D intakes and mean serum 25-hydroxyvitamin D concentration in Korea, 2009, by age and gender.

Gender	Age (Years (*n*))	Energy Intake (kcal/Day)	Vitamin D Intake (µg/Day)		AI (µg)	Less than AI (% (*n*))	Serum 25(OH)D (ng/mL)	25(OH)D Deficiency Ratio (% (*n*))
			Unadjusted	Adjusted for Energy				
Men	20–29 (281)	2380.76 ± 63.94	3.72 ± 0.42	3.67 ± 0.41	5	80.1 (225)	16.43 ± 0.50	76.8 (208)
	30–49 (798)	2455.26 ± 31.62	4.72 ± 0.28	4.59 ± 0.28	5	74.6 (595)	18.76 ± 0.39	64.2 (496)
	50–64 (555)	2238.57 ± 41.07	4.29 ± 0.33	4.42 ± 0.33	10	90.5 (502)	20.92 ± 0.40	48.7 (257)
	65–74 (290)	1970.43 ± 60.34	3.70 ± 0.35	4.17 ± 0.33	10	90.3 (262)	21.16 ± 0.55	49.0 (133)
	≥75 (97)	1719.45 ± 70.69	2.38 ± 0.43	3.16 ± 0.49	10	95.9 (93)	21.65 ± 0.80	40.9 (39)
	Total (2021)	2152.89 ± 28.03	3.76 ± 0.19	4.00 ± 0.17			19.78 ± 0.33	61.8 (1133)
	*F*-value	30.149 ***	6.270 ***	2.376			23.522 ***	
Women	20–29 (298)	1724.42 ± 50.40	2.48 ± 0.17	2.42 ± 0.17	5	86.6 (258)	14.71 ± 0.35	86.1 (258)
	30–49 (1074)	1703.78 ± 20.37	3.02 ± 0.15	2.98 ± 0.15	5	84.5 (907)	16.07 ± 0.28	78.8 (829)
	50–64 (666)	1655.69 ± 30.78	2.97 ± 0.24	2.97 ± 0.23	10	93.8 (625)	17.92 ± 0.29	67.7 (441)
	65–74 (350)	1435.24 ± 34.18	2.25 ± 0.22	2.47 ± 0.21	10	96.3 (337)	18.48 ± 0.50	62.7(208)
	≥75 (132)	1374.52 ± 62.41	2.10 ± 0.34	2.39 ± 0.35	10	95.5 (126)	18.32 ± 0.69	64.0 (85)
	Total (2520)	1578.73 ± 19.41	2.56 ± 0.10	2.64 ± 0.10			17.10 ± 0.26	75.4 (1821)
	*F*-value	17.331 ***	3.668 **	2.302			16.741 ***	

Data are expressed as means ± standard errors, except for “25(OH)D deficiency ratio” and “Less than AI”, which are expressed as percentages (numbers). The cut-off point (deficiency level) for serum 25(OH)D concentration was 20 ng/mL. *F*-values were based on the results from the complex samples general linear model. ** *p* < 0.01; *** *p* < 0.001; AI, adequate intake; 25(OH)D, 25-hydroxyvitamin D.

**Table 3:** Relationship between vitamin D intake and serum 25-hydroxyvitamin D concentration adjusted for confounding factors.

Subjects	Gender (*n*)	β	95% CI	R^2^	*F*-Value
Total subjects (*n* = 4541)		0.044	0.009~0.079	0.089	6.196 *
<50 years old	Total (2451)	0.049	0.005~0.093	0.072	4.837 *
	Men (1079)	0.016	−0.039~0.071	0.094	0.328
	Women (1372)	0.06	−0.003~0.012	0.02	3.571
≥50 years old	Total (2090)	0.015	−0.043~0.072	0.059	0.25
	Men (942)	−0.017	−0.088~0.054	0.046	0.234
	Women (1148)	0.052	−0.050~0.154	0.046	1.01

Confounding factors; age, drinking status, smoking status, physical activity-high, sun exposure time and use of vitamin/mineral supplements. *F*-values were based on the results from the complex samples general linear model; * *p* < 0.05; CI, confidence interval.

**Table 4:** Contribution of vitamin D-rich food groups towards the daily mean intake of vitamin D in Korean adults.

Food Groups	Total	Vitamin D Intake (Contribution)		*F*-Value
	( *n* = 4541)	Man ( *n* = 2021)	Women ( *n* = 2520)	
Fish & shellfish	2.42 ± 0.10	2.81 ± 0.17 (71.69)	2.02 ± 0.10 (62.35)	13.790 ***
Eggs	0.67 ± 0.02	0.68 ± 0.03 (17.05)	0.65 ± 0.03 (20.05)	0.691
Milk & dairy products	0.20 ± 0.01	0.16 ± 0.02 (4.12)	0.24 ± 0.02 (7.41)	6.146 *
Meat & meat products	0.14 ± 0.02	0.12 ± 0.01 (3.22)	0.17 ± 0.03 (5.25)	3.336
Mushrooms	0.13 ± 0.02	0.11 ± 0.02 (2.94)	0.13 ± 0.03 (4.01)	0.2
Grain products & others	0.04 ± 0.00	0.04 ± 0.01 (0.98)	0.03 ± 0.01 (0.93)	0.575

Data are shown as means ± standard errors, and adjusted for energy intake. Contribution is also expressed as percentages (%). *F*-values were based on the results from the complex samples general linear model. * *p* < 0.05, *** *p* < 0.001.

**Table 5:** Daily and accumulated contribution of vitamin D-rich foods in Korean adults.

Rank	Men ( *n* = 2021)			Women ( *n* = 2520)		
	Food	Con (%)	Acc-con (%)	Food	Con (%)	Acc-Con (%)
1	Anchovy	23.58	23.58	Anchovy	23.91	23.91
2	Mackerel	15.08	38.66	Egg	14.61	38.52
3	Egg	13.74	52.4	Mackerel	14.17	52.69
4	Bastard halibut	7.34	59.75	Pacific saury	6.26	58.96
5	Pacific saury	6.41	66.16	Milk	5.59	64.55
6	Hairtail	4.81	70.97	Hairtail	4.97	69.51
7	Eel	3.52	74.49	Bastard halibut	4.28	73.8
8	Milk	3.07	77.56	Eel	3.37	77.17
9	Chum salmon	2.1	79.67	Chum salmon	2.22	79.39
10	Tuna	2.09	81.75	Oak mushroom	2.04	81.43
11	Pork	1.79	83.54	Jew’s-ear	1.91	83.35
12	Loach	1.69	85.23	Filefish	1.88	85.23
13	Filefish	1.57	86.79	Tuna	1.83	87.06
14	Jew’s-ear	1.49	88.28	Pork	1.72	88.78
15	Yellow croaker	1.3	89.58	Shrimp	1.11	89.89
16	Oak mushroom	0.97	90.55	Yellow croaker	1.09	90.98
17	Puffer	0.77	91.32	Loach	0.83	91.81
18	Shrimp	0.76	92.07	Spanish mackerel	0.74	92.54
19	Seabream	0.74	92.82	Angler	0.67	93.21
20	Spanish mackerel	0.72	93.53	Oyster mushroom	0.65	93.86
21	Trout	0.51	94.04	Alaska pollock	0.53	94.39
22	Angler	0.49	94.54	Puffer	0.44	94.82
23	Cereal	0.45	94.98	Common Mullet	0.4	95.22
24	Gizzard shad	0.43	95.42	Gizzard shad	0.4	95.62
Total		95.42			95.62	

Con, contribution; Acc-con, accumulated contribution.
